# Seventh Conference of the Association of Hospital Superintendents

**Published:** 1905-10-28

**Authors:** 


### SEVENTH CONFERENCE OF THE ASSOCIATION OF HOSPITAL SUPERINTENDENTS.

### AMERICAN VIEWS AND OPINIONS.

(*Continued from page* 58.)

### Medical and Hospital Libraries.

Dr. George H. M. Rowe, President of the Conference, then announced the recent sudden death of Dr. J. R. Chadwick, the founder of the Boston Medical Library, who had been expected to deliver a paper under the title of "Medical Libraries in Hospitals." Dr. Rowe gave a short sketch of the life of Dr. Chadwick, concluding with an admiring tribute to the man whose energy and perseverance had built up such a much-needed institution as the Boston Medical Library. Dr. Edward H. Brigham, Dr. Chadwick's assistant, read a paper prepared by his former chief on the founding and description of the library. "Some Details About Libraries in Hospitals" was the subject of an interesting paper by Mrs. Grace Whiting Myers. She maintained that a medical library is a necessity in a hospital. It is ready for emergencies, for study and for rest, for meetings and discussions. There are few real working libraries, for the need for them is not realised. In the management, she recommended the most simple method of cataloguing and maintained that to the librarian belongs the success of the medical library.

Dr. Hurd opened the discussion of this paper with the remark that he thought the necessity for a library in the hospital is self-evident and he also recommended a large library for nurses, since the wider the horizon of a nurse, the better her grasp of her profession. Sir Henry Burdett maintained that to do efficient work it is essential to consort with books. There in the biographies of the world one finds consolation and the strength to go forward. Never deprive or divorce us from the companionship of books. Sir Henry as representing The Hospital in journalism wished to join hands with America. "The field," he says, "is large enough if we co-operate to make a journal and a year-book of permanent interest. It is not so much a matter of finance as of co-operation."

### Multiple-Storied Hospitals in Cities.

Dr. G. H. M. Rowe opened the Session by announcing that Dr. Ochsner, Surgeon-in-Chief of Augustana Hospital and St. Mary's Hospital of Chicago, who could not be present at the time assigned for his paper, had arrived, and would now read that paper. Dr. Oschner's subject was "Multiple-Storied Buildings for Hospitals in Cities." It is his opinion that for cities like Boston, New York, and Chicago, and large cities generally, the many storied plan is best. He gave the requisites for an ideal hospital as (1) Sunlight in every room or ward of the building; (2) proper heating; (3) proper conditions for ventilating; (4) safe disposition of sewerage; (5) absence of noise in the hospital.

To these may be added (6) safety from fire; (7) relative isolation of patients when desired; (8) possibility of increase in the future.

He looks upon the question of economy as of great importance, since money spent in the actual construction of the building is the least part of the expense. The conduct of the hospital is on the expensive side, therefore the hospital structure itself must be built to lessen these running expenses as much as possible. In the multiple-storied building, just as in a low structure, much of the ventilation must come through open doors and windows, therefore a building standing in an open space has a better circulation than a number of small buildings standing close together in that same space. There is also advantage to be obtained from getting away from the earth, therefore the multiple-storied building has the advantage of ventilation and sunlight over the pavilion style. More than one-half the buildings on the cottage plan are contaminated with street dust, while in the higher stories of the tall building this is not the case. Artificial ventilation by fans can be accomplished as readily in the multiple-storied building as the pavilion style. The heating should be supplied by direct radiation, both because it is more hygienic and less expensive than indirect. The absence of noise in the hospital is important, and the amount of street noise which will reach patients on the sixth story is much less than on the first or second. In a hospital where the recovery-rooms are on the top floor, and also the operating rooms one secures all the requisite facilities and at the same time has the advantage of avoiding dust and noise. Dr. Ochsner proved conclusively that in a ten-story building one could obtain the same facilities at one-half the cost of construction of a hospital of the same number of beds constructed on the pavilion plan. Relating to a home for nurses he recommended that in the young days of the hospital they be housed on one floor of the main building, but that ultimately they have a separate house, since it was better for their health and they could render better service.

### Hospital Engineering and Hospital Waste.

Under the general topic of "Hospital Engineering Problems" Dr. Renwick R. Ross, Chairman of the Session, Dr. John H. McCollom, Dr. F. A. Washburn, and Mr. Jeremiah C. Long, each read papers. Dr. Ross's subject was "Cold Storage and Refrigeration in Hospitals." He said that the best things for cold storage were wool and absorbent cotton, but as these were too expensive for general use he recommended still air as the next best thing; and ammonia or brine system are the only methods applicable to a general storage plant of the size required in an institution. In the preservation of food the chief factor is the temperature, but of almost equal importance are clean, dry, well-ventilated rooms, and pure air. To employ cold storage successfully depends upon good judgment and good food. There is a great advantage in employing this method because of the saving of money and of the greater variety of food obtainable when cold storage is judiciously employed.

Dr. John H. McCollom gave a paper on "Refuse Destroyers and Disinfectants." He emphatically stated that the hospital is not a focus for the spread of diseases, since all refuse matter is burned on the premises. The expense of cremation can be made small by using the heat, generated by the combustion of refuse, for the manufacture of steam. Considering the amount of refuse destroyed, the expense is very slight and the furnace is justified in its results. Steam uunder pressure is the best disinfectant for mattresses, clothes, and also patients. Formaldehyde gas is effective as a disinfectant since it does destroy animal life, but it has no penetrating powers. Corrosive sublimate is fine for the walls and floors of a hospital; chloride or zinc cannot be relied upon to destroy micro-organic life.

In the case of infectious diseases, with the exception of small-pox, Dr. McCollom said that if the patient was removed from the general ward at an early stage of the development of the trouble it was not necessary to disinfect the ward except in the immediate vicinity where the patient had been.

"The Utilization of Hospital Waste" was the subject of a paper by Dr. F. A. Washburn of the Massachusetts General Hospital. He demonstrated what the hospital had done in the way of utilising their waste gauze. All the gauze now used in the wards is used first in the operating room; then put through a process of resterilization, taken to the wards and used there for dressings, thus instituting a system of economy by using material formerly considered waste. After discussion, it was proved by Dr. Washburn, to the satisfaction of the members, that the process of re-sterilization was a perfectly safe and legitimate one.

The paper read by Mr. Jeremiah C. Long, member of Society of Mechanical Engineers and chief engineer of the Boston City Hospital, on "Engine-room Economies" dealt chiefly with the question of leakage.

The discussion was led by Mr. Clarence W. Williams, chief engineer of the Massachusetts General Hospital. He recommended a combination of the high and low steam pressure in the matter of heating.

### The Uniform System of Accounts.

The general subject of the afternoon meeting was "Hospital Accounts." The first paper was read by Dr. C. Irving Fisher, Presbyterian Hospital, New York, N.Y. Chairman of the Session. He asked for a common plan on which hospital accounts could be drawn up. He said that the accounts must be grouped against the work done and must show the relationship between this work done and the money expended. He presented a tentative schedule for hospital accounts.

Dr. James R. Coddington, New Haven Hospital, Conn., spoke on the same subject. He is greatly in favour of the report submitted, but this schedule must be thoroughly talked over and thought about before being generally accepted.

Miss Maud Banfield read a paper on "Hospital Statistical Tables as to Classification of Patients."

Sir Henry C. Burdett led the discussion and explained the advantages of a uniform system of accounts. After some debate the Conference appointed a Committee with power to employ an experienced accountant. It was decided to devote the next year to promulgating the uniform system throughout the United States.

### Pensions and Old Age Provision.

"The Royal National Pension Fund" was the subject of Sir Henry Burdett's address on Friday. In a review of the growth of the National Pension Fund, he showed that the fund was meeting with the endorsement and co-operation of nurses throughout England. In 1891 he had attempted to inaugurate a similar fund in America but did not meet with any marked success because the nurses in America did not fall into line.

Sir Henry paid a tribute to the excellent spirit that pervaded this Seventh Conference of the Association of Hospital Superintendents, and he thought that the Conference would send forth new energy and light to every hospital in the United States. "Render personal service gladly, not because you must but because you will. Put on the Christ life. Try and follow that, and let it be the beacon light for us, who so often must witness sickness and suffering and the ravages of disease. Sow, sow, sow, personal service."

Buffalo was selected as the place for the next meeting.

A Committee, composed of Dr. J. O. Skinner, Dr. C. I. Fisher, and Mr. James R. Coddington, was appointed to work out a scheme for uniform accounts.

### Question Box Conducted by John M. Peters, Chairman of the Session.

*Q.*—Should visitors be admitted to the wards in the evening?—Drs. Ross and Fisher took the affirmative on the basis that certain classes of working people could only visit their friends and relatives during evening hours. Dr. Rowe considered that too much visiting is a mistake, since it interferes with the duty to the patient.

*Q.*—What are the best floors for operating rooms and wards?—Dr. Henry M. Hurd claimed that maple is best for ward floors, but since it is unduly slippery Southern pine can be used with almost as good results. He recommended a linoleum covering for the floors as it deadens noise. For the operating-room he suggested heavy hexagonal tiles laid with great care.

*Q.*—Is it advisable for officers and pupil-nurses to eat at the same table and have social intercourse when off duty?—It was generally agreed that there must always be a distinct demarcation between the officials and the employés of an institution; therefore, except on special occasions, this relationship should be sustained.

*Q.*—What is the best method of conducting laundry against cost and loss of material?—The solution of this question is competent oversight in the ward and in the laundry.

*Q.*—Shall pupil-nurses be paid?—Dr. Ross, of Buffalo General Hospital, argued in the negative and said that in his hospital the money which had been used heretofore to pay the nurses was now spent in obtaining better and more instructors and in giving the nurses advantages along educational lines which had been impossible before.

